# Medical News and Miscellany

**Published:** 1894-01

**Authors:** 


### Medical News and Miscellany.

**Dr. R. P. Tye** has removed from Clarendon, Texas, to Chickasha, Indian Territory.

---

**Dr. H. B. Granberry** has been appointed local surgeon of the I. &. G. N. R. R. at Austin, Texas.

---

**Dr. E. E. Johnson** has removed from Ladonia to Denison, and will make a specialty of diseases of women and children.

---

**Married,** December 22, 1893, at Marble Falls, Texas, by Rev. Chas. Daniels, Dr. J. R. Yett to Miss Ollie Phillips, all of Marble Falls. THE JOURNAL extends happy new year and congratulations to the young couple.

---

**A Fire at Decatur, Texas,** on 22th of December, ult., destroyed much property, including the office and contents of our friends Drs. York & Miller. They had $4500 insurance, however, and we hope, are not not much hurt.

---

**Married.**—At Burton, Texas, December 27th, ult., Mr. W. C. Manlove, of Burton, to Miss Ethel Hons, daughter of Dr. J. M. Hons, of San Marcos, Texas. [We believe the groom is a son of the gallant Col. Tom B. Manlove, of Vicksburg.—ED.]

---

**Dr. W. F. Waugh** has thrown up the sprightly *Times-Register*, given up all his positions in Philadelphia, and removed to Chicago! There is something, must be, irresistible in the attractions to Chicago when one fixed like Waugh, lets go to go there. We wish him luck.

---

**The S. W. Cockers' Journal,** "the sportsman's favorite," for December, is out. It is a very sprightly periodical, and every breeder of fine stock or fancier of game birds, or lover of the rod and gun, should subscribe for it. Published at San Antonio,

Texas, by Mr. F. V. Daniel, breeder of the finest strains of game birds. $1.00 a year.

---

**Hymeneal.**—The JOURNAL is in receipt of cards announcing the marriage, to take place on 11th of January, inst., of Dr. J. M. Richmond to Miss Marie Bronaugh, all of Edna, Texas. Dr. Richmond is a son of Dr. W. T. Richmond, of Manor, and is a graduate of Marion-Sims Medical College of the Class of '92-3. Happy New Year!

---

**Dr. H. W. Waters,** of Brenham, the oldest resident physician of that county, and a prominent member of the Texas State Medical Association, was killed on the streets of Brenham on the 4th of January, inst., by a stray bullet. He was hastening to separate two lads who were fighting, when one of them fired a pistol, with the result stated.

---

The *Texas Sanitarian* has reduced its subscription price to $1, but says the publication will not be "cheapened" correspondingly, but that the same high standard of excellence will be maintained. This enables us to offer it as a premium to our subscribers, our club rate being $2.50 for the two journals to all new subscribers. Begin with January, 1894.

---

**Wanted.**—Accurate or approximate information on the following points: How many regular practicing physicians are there in Texas? How many are members of Medical Societies—local or district? How many belong to no such organization? Any one who can furnish the above information will confer a favor by communicating it to Dr. J. H. Sears, President Texas State Medical Association, Waco, Texas.

---

**Funny Bone.**—We have received a copy of "*Funny Bone.*" We are a little disappointed. Some jokes not particularly good, appear in several places in a little different garb; too many paragraphs taken from *Pharm. Era.* Though, it *is* certainly worth 50 cents, if only for the pictures, some of which are funny. We do not exactly see where the "bone" comes in; it is not a bone, it is a book, and bears no resemblance to a bone. We don't see the connection.

---

We see some of the journals are "pitching in" to Dr. Gould on account of his peculiar spelling, and his use of words that are not in any dictionary but his. There is need for pruning in the use of some words; for instance, "programme," "catalogue," etc.; but Dr. Gould makes new words, it seems, and some people object to them. We no longer speak the English language; why not have a dictionary of "United States?" We observe in Webster's latest, the words "sockdolager" and "skeedaddle"; why blame Gould for his departure?

---

**Hon. Dudley G. Wooten.**—We are promised a paper for our next issue from the pen of Hon. Dudley G. Wooten, of Dallas, on a subject which is now attracting much attention all over the United States: "Sexual Crimes in Relation to Jurisprudence." He will take Dr. Daniel's paper on "Sexual Perverts and Castration," published in the December number of the TEXAS MEDICAL JOURNAL, and the *Medico-Legal Journal*, of New York, for his text, and will treat the subject from the standpoint of criminal law. That it will be an able and well written article, "goes without saying,"his well known ability both as a lawyer and a writer being ample warrant for the assertion.

---

**Hard on the Employees.**—The following notice is posted in the pension office at Washington:

"Members of the medical division are forbidden to have their hats or clothing on prepartory to leaving this office before 4 o'clock. Any one breaking this rule will be charged with a demerit of 15 minutes."

It is perhaps not strictly our business, but we should think it would be rather uncomfortable for the clerks of the medical division to work all day without any clothing.—*New York Tribune.*

Such an order would make one believe that the pension employees are decidedly "in the swim." It would be more appropriate on the door of a natatorium than on a pension bureau.

This reminds us of Pat's reply when the Jew wanted to "sell him a trunk to put his clothes in." "What," said Pat, "and go naked?"

---

**A letter** of the Secretary General's, dated November 29th, informs me that "traveling documents" will be sent to the address of every subscriber on or before February 15, 1894, and that after that date congressists will have to apply to the undersigned.

It also contains the following regulations of former circulars:

Members' dues are five dollars (money order to Prof. L. Pagliani, Rome), guests' (wives and adult relations) two dollars, medical students: no fees. All are entitled to traveling documents.

Reductions on the Italian railways are available from March 1st until April 30th. A. JACOBI, M. D.,

Chairman Nat. Committee International Med. Congress.

---

**Special Notice.**—This is not only "special" notice, but "general" notice, and "important" notice. The TEXAS MEDICAL JOURNAL feels the "stringency" as well as other people (we are "people"), but out of consideration of the "rocky" times and sympathy with our fellow sufferers and fellow sinners, we have held up our bills for subscription, pressing none for payment. The beginning of a new year is here now, and our patrons have gotten in their collections, if they will ever get them in; and having sent out every bill on our books, so that no one will consider himself especially "dunned," we respectfully and *earnestly request* payment, at least in part, from every one in arrears who can, without too much inconvenience to *their* "Betty and the Baby," divide with us. We are just "obleeged" to do it. Can't keep up all this swell style—a hundred pages, red hot, every month—without a "leetle" money; we have to pay cash on delivery for every issue. We trust our good friends will appreciate the situation, and generously respond, as they have always done when called on. We are going to publish a list of all who answer this, in the order in which remittances are received, but not amounts, because some are away in arrears, and if they will pay up, we will say nothing about it, but will remember them when we are distributing our blessings, and they will feel better. All aboard—let her go, Johnny.

P. S.—Silver, greenbacks, express orders, bank drafts and money orders taken without discrimination or discount.

---

**Kind Words.**—The TEXAS MEDICAL JOURNAL for December, is to hand promptly on time, and we can say without hesitation or mental reservation that it surpasses in mechanical makeup, and in the matter of its contents any that have preceded, and we have read the JOURNAL from the first number ever issued. Dr. Daniel, its editor, like good wine, seems to be growing better with age. He did not give us his best at the beginning of the feast.—*Lampasas Leader.*

THE TEXAS MEDICAL JOURNAL.—We always turn at once to the editorial notes of this journal. It is a journal that is not only bright without, but still brighter within. Published at Austin, Texas, we notice in the last number that the editor invites all of his best friends to call upon him when they visit the "city of the violet crown." Dr. Daniel knows what to say on every subject that he discusses, and he knows just how to say it too. Among the original communications in the last number, we note the following: A case of Tubercular Meningitis; Congenital Anchylosis of sixteen Joints; Teaching Anatomy in Medical schools; Polio-Myelitis; together with some interesting correspondence. "Our Wile(y) friend of the Bile-colored *New England Journal,*" comes in for a most caustic editorial. Dr. Daniel republishes, by request, one of his original poems, entitled, "The Doctor's Lament." It is a story of the earnest pleadings of a doctor to his lady-love. The journal is published monthly and the subscription price is $2 a year. If anybody subscribes for this journal and does not get his money's worth in reading the editorial notes alone, we will give him a year's subscription to this journal free.—*December Number of "Food."*

---

**State Lunatic Asylum, Austin.**—The annual report of Superintendent White of the lunatic asylum for 1893 shows that the institution has been well managed and is very prosperous. During the year 121 patients were admitted, 43 discharged restored, 3 discharged improved, 3 discharged unimproved, 1 escaped, 27 died, leaving on hand October 31, 624, of whom 357 were males and 297 females.

Of those admitted during the year 70 were males and 50 females, 111 whites and 10 colored. Of the number 61 were married, 46 single, 6 widows, 2 widowers, 6 unknown. Of the whole number admitted 39 were farmers, followed by housewives 29. Of the cause of insanity of those admitted, heredity heads the list and is put down at 30, intemperance 9, and religious excitement 5. Since the beginning of the asylum there have been admitted to it 2380, patients discharged 1944, died 725, on hand 654.

Dr. White mentions the improvements made, and underway, and highly compliments Drs. Worsham and Maxwell, Bookkeeper S. M. Torbett, Matron Brannon and others.

The board of managers compliments Dr. White and says:—"While not wishing to flatter Dr. White, we can say that, as superintendent, we have always found him loyal to his trust in every particular, and that the degree of harmony prevailing between the superintendent and the board of managers is marked.''

The bookkeeper reports as follows:—By reference to exhibits 1 and 2, it will be seen that the total expenditure for the year is $167,432.70. Of this amount $68,890.84 was for permanent improvements, repairs. stock on hand, etc., per tabulated statement, leaving a balance of $98,551.86 for actual maintenance,

The number in daily attendance was 623 and results in an annual cost of $158.18 per capita.

The value of farm products raised during the year was $4095, and garden truck $2154.

### IN MEMORIAM.

At an informal meeting of Johnson County Medical Association, held at Cleburne, Texas, December 21, 1893, the following resolutions were adopted:

*Mr. President and Gentlemen:*—As a committee appointed to draft suitable resolutions on the death of Dr. J. L. Wagley, we respectfully beg leave to submit the following:

WHEREAS, Death has ruthlessly stepped in and removed from among us our worthy friend and coworker, Dr. J. L. Wagley, we pause for a moment and stagger to think of the awful calamity and sad ending of our president and friend, and to express and extend our sympathy to his bereaved family; therefore, be it

*Resolved*, 1. That in the death of Dr. Wagley we lose our most worthy president and coworker.

2. We are denied the pleasure of his association, both as a physician and friend, in all the pleasures and ills that pertain to humanity.

3. His loss to our Association and to the community will be keenly and strongly felt, both by his coworkers and the people at large.

4. That in his untimely death we feel a bitter sadness that is hard to overcome, and that we, as his brother physicians, express and extend to his family our profoundest sympathy and deepest regrets.

5. That these resolutions be spread upon the minutes of this Association, and a copy sent to the papers for publication, also a copy sent to TEXAS MEDICAL JOURNAL, and a copy be sent to his family.

Respectfully submitted,

W. P. ALEXANDER, M. D.,
J. D. OSBORN, M. D.,
J. J. WILLIAMSON, M. D.

---

### RAILWAY SURGERY AND THE MEDICO-LEGAL SOCIETY.

The proposal to found a section upon Railway Surgery in the Medico-Legal Society, composed of railway surgeons and railway lawyers, met with the approval of that body.

The section will be managed by a chairman and ten vice-chairmen from each profession of law and medicine, a secretary, and a treasurer.

Every member of the Medico-Legal Society, ingood standing, is eligible to membership in the section without entrance fee and nominal dues of $1.50 per annum.

Every railway surgeon or railway lawyer is eligible to membership on payment of $5 and annual dues of $2 each, who is not a member of that body, or he can unite with the society and become a member of the section.

The selection of the chairman and vice-chairmen from both professions will be fixed at the conference during the coming fall months.

The railway surgeons of the United States and the Canadas are invited to unite with the Medico-Legal Society and with the section.

The initiation fee to the Medico-Legal Society is $5, and the annual dues, $2 each year, outside the State of New York, and the *Medico-Legal Journal* will be sent free to each member of the section in good standing, free of charge.

Among the prominent members of the National Association of Railway Surgeons who have united with the Medico-Legal Society, and who will take a prominent position in the work of the section, on its medical side, are: Drs. Granville P. Conn, of New Hampshire; B. F. Eads, of Texas; R. Harvey Reed, of Ohio; Nicholas Senn, of Illinois; W. J. Gabrath, of Omaha, now president of that body; W. B. Outten, of St. Louis; George Chaffee, of Long Island R. R,; R. S. Harnden, of Waverly, N. Y.; Richard J. Nunn, of Savannah; A. M. Phelps, of New York; Thomas H. Manly, of New York; Matthew D. Field, of New York; S. Grover Burnett, of Kansas City, and a large number of the medical men of the society will identify themselves with its labors. If two or three hundred of the more enterprising members of the National Association of Railway Surgeons this fall unite with the section, and leading railway sounsel also take similar action, the labors of the body will be of inestimable service to the advance of scientific knowledge in the future—*Medico-Legal Journal*, Sept. '93.

Among the large number of Railway Surgeons and Railway counsel elected to membership in the Medico-Legal Society at its October meeting (1893) we find the names of the following Texas physicians:

Dr. W. H. Monday, Terrell, Tex., chief surgeon Texas & Midland R. R.

Dr. C. A. Smith, Tyler, Tex., chief surgeon St. Louis ——— R. R.

Dr. W. H. Dailey, Paris, Texas, local surgeon Texas & Pacific R. R.

Dr. G. P. Howard, Marshall, Texas, surgeon Texas & Pacific R. R.

Dr. B. F. Eads, of Marshall, is a member, and is honored by the publication in the *Medico-Legal Journal* of his biography, and his picture, which appears in a group of distinguished railway surgoons. Dr. D. R. Wallace is vice-president for Texas, of the Medico-Legal Society, and honorary vice-president, as were Dr. F. E. Daniel, of Austin, and Hon. G. W. Tyler, of Belton, of the late International Medico-Legal Congress held in Chicago.

The Medico-Legal Society was organized in New York in 1862, with a handful of members, and has grown to international importance, numbering among its members many of the most distinguished medical and legal men in America, Europe, South America, Mexico and Canada,—and having members and vice-presidents in every State and Territory of the American Union, and in many foreign States and countries.

Apropos of the comparatively little interest taken by American officials and physicians in the Medico-Legal Congress referred to above, held in Chicago in August last, Dr. M. Louise Thomas, of New York, during the session—amongst many other important andinteresting things, said:

"I don't know whether President Bell would like me to refer to an experience I had last summer when the Congress of Criminal Anthropology was held in Brussels. The Medico-Legal Society honored me by sending me as a delegate to that congress, and I also represented the International Medico-Legal Congress as a delegate. It was a most remarkable gathering of leading scientists of the world, under the direct patronage of the King of Belgium, Leopold II. Nearly all the delegates came under the direct authority of the general government of Belgium. They were most upright and honorable gentlemen; their papers strong stirring, and original. Some of them were very unique, as you will find by reading the proceedings. They went into branches of thought never before fully measured. The subject of heredity was one; measurement of the cranium, another, and some other subjects new and interesting to me. The deliberations were all in French, the court language of that part of the world. The King of Belgium honored the occasion by his presence in the hall, and, still further, by giving the congress a reception at his palace. When the delegate from America was presented to him, he expressed his great pleasure in receiving a delegate from the

International Medico-Legal Society from the United States. He also expressed the wish and hope that he might be with us on this occasion, and during our experience to-day I have wondered whether, if King Leopold had honored the occasion with his presence, our own people would recognize still more the importance of the subject that has brought us together. I have not time now, I know I must not take more time, but if, during the congress I should have opportunity to speak, I shall be glad to further enlarge upon what I have merely suggested."

